# SARC-F as a Screening Tool in Rheumatoid Arthritis: Real-World Burden of Sarcopenia Risk, Sex Differences, and Clinical Correlates

**DOI:** 10.3390/jcm14217751

**Published:** 2025-10-31

**Authors:** Joan M. Nolla, Lidia Valencia-Muntalà, Laura Berbel-Arcobé, Diego Benavent, Paola Vidal-Montal, Martí Aguilar-Coll, Montserrat Roig-Kim, Javier Narváez, Carmen Gómez-Vaquero

**Affiliations:** Department of Rheumatology, IDIBELL-Hospital Universitari de Bellvitge, University of Barcelona, 08029 Barcelona, Spainfjnarvaez@bellvitgehospital.cat (J.N.); carmen.gomez@bellvitgehospital.cat (C.G.-V.)

**Keywords:** rheumatoid arthritis, sarcopenia, SARC-F, sex differences, patient-reported outcomes

## Abstract

**Background/Objectives:** Sarcopenia is now recognized as a frequent and disabling accompaniment of rheumatoid arthritis (RA), although structured screening approaches are still rarely applied in everyday practice. The SARC-F questionnaire offers a simple, validated, patient-reported tool for sarcopenia screening, but its performance in RA remains largely unexplored. We aimed to evaluate the burden of sarcopenia risk, defined by abnormal SARC-F scores (≥4), and its clinical correlates in RA compared with age- and sex-matched controls. **Methods:** We conducted an observational case–control study including 275 RA patients (69.5% women) aged >50 years and 300 matched controls. Clinical, laboratory, and patient-reported outcomes were recorded. Sarcopenia risk was assessed using SARC-F (cutoff ≥ 4). RA patients also underwent grip strength and gait speed testing. Multivariable regression analyses were used to identify independent correlates of abnormal SARC-F results. **Results:** A SARC-F score ≥ 4 was observed in 26.9% of RA patients. Compared with controls, the burden was significantly higher in women with RA (34.0% vs. 24.7%, *p* < 0.05) but not in men (10.7% vs. 15.0%). Within the RA cohort, abnormal SARC-F was independently associated with female sex (OR 3.14, 95% CI 1.24–7.95) and higher RAPID3 scores (OR 1.25, 95% CI 1.18–1.33). More than half of RA patients exhibited low grip strength, with partial overlap with SARC-F findings. **Conclusions:** The SARC-F questionnaire revealed a notable burden of sarcopenia risk in RA, particularly among women. Combined with simple grip strength testing, it offers a feasible, low-cost approach to case finding, directly applicable in routine rheumatology practice. Incorporating this strategy may enhance recognition and management of sarcopenia in RA.

## 1. Introduction

Sarcopenia [1] refers to a decline in muscle quantity and function that contributes substantially to physical impairment, reduced quality of life, and an increased burden of adverse health outcomes in chronic disease. In rheumatoid arthritis (RA), the combined effect of persistent systemic inflammation, chronic pain, and reduced physical activity creates an environment that favours secondary sarcopenia [2], with the potential to amplify disability and compromise treatment outcomes. In this context, identifying sarcopenia in patients with RA is clinically meaningful, as it signals a vulnerable functional phenotype that may worsen long-term prognosis and is amenable to targeted intervention.

Over the past decade, the incorporation of patient-reported outcome measures (PROMs) has transformed the assessment of RA impact [3]. The SARC-F questionnaire [4] is the principal brief, equipment-free PROM recommended by the European Working Group on Sarcopenia in Older People (EWGSOP2) [5] as a first-line case-finding tool. However, despite its suitability for high-throughput clinical settings, little is known about its performance in routine rheumatology practice or its value in guiding clinical decision-making. Consequently, it remains unclear whether systematic implementation of SARC-F in RA is effective, which patient profiles might benefit most, and to what extent its results align with relevant clinical and functional parameters.

To address this gap in real-world evidence, we conducted an age- and sex-matched case–control study to quantify the burden of sarcopenia risk in RA compared with individuals without inflammatory arthritis, using SARC-F as the screening tool. In addition, we sought to explore potential sex differences and to identify the clinical correlates of SARC-F scores within the RA population, with the ultimate goal of informing more targeted and patient-centred screening strategies in rheumatology practice.

## 2. Method

### 2.1. Study Population

We performed a case–control study that enrolled individuals over 50 years of age. Patients with RA were included consecutively during routine outpatient visits at our tertiary rheumatology centre, provided they met the 2010 ACR/EULAR classification criteria. Controls were selected from a hospital-based population without inflammatory arthritis and matched to RA cases by sex and age. Each patient was paired with controls of the same sex and similar age, but no matching was performed between men and women with RA. Control participants were enrolled from three sources: accompanying relatives of rheumatology outpatients, individuals with non-inflammatory musculoskeletal complaints (predominantly soft-tissue disorders), and hospital attendees presenting for non-musculoskeletal reasons. All participants were consecutively screened using the same predefined criteria.

To minimize confounding, both cases and controls were excluded if they had conditions associated with secondary sarcopenia (active malignancy, heart or respiratory failure, chronic liver disease, or chronic kidney disease). Other common comorbidities not associated with sarcopenia, such as hypertension or dyslipidaemia, were not considered exclusion criteria and were not systematically recorded. The same selection process and exclusion criteria were applied to cases and controls to reduce selection bias. All individuals gave written informed consent before inclusion, and the study was conducted under the approval of the local ethics committee (PR057/20).

### 2.2. Study Variables

#### Demographic and Anthropometric Characteristics

These variables were recorded for both RA patients and controls. We included the following:

- Sex.
- Age.
- Body Mass Index (BMI): Calculated as weight (kg) divided by height squared (m^2^). Participants were classified as
  ▪ Underweight: <18.5 kg/m^2^;
  ▪ Normal weight: 18.5–24.9 kg/m^2^;
  ▪ Overweight: 25–29.9 kg/m^2^;
  ▪ Obese: ≥30 kg/m^2^.

- Smoking Status: Categorized as never smokers, current smokers, or former smokers.
- Physical Activity: Defined by self-reported frequency and intensity:
  ▪ None;
  ▪ Occasional;
  ▪ Regular, low-intensity;
  ▪ Regular, high-intensity.

### 2.3. Hemoglobin Level

Hemoglobin level was assessed in both RA patients and controls, using the most recent available value for each participant. For RA patients, laboratory tests corresponded to the most recent assessment within the previous 3 months, coinciding with the clinical visit in which SARC-F and functional measures were obtained. In controls, laboratory values were taken from the most recent routine analysis available, with no relevant clinical changes expected between assessments.

### 2.4. RA Assessment

Clinical history and serology. We documented key RA characteristics, including disease duration, seropositivity for rheumatoid factor (RF) and anti-citrullinated peptide antibodies (ACPAs) with their respective titers, and current pharmacotherapy—namely, glucocorticoids, conventional synthetic DMARDs, biologic DMARDs, and Janus kinase inhibitors, all recorded as current treatment at the time of assessment.

- Laboratory Parameters. The most recent blood tests were reviewed for erythrocyte sedimentation rate (ESR) and C-reactive protein (CRP).
- Disease Activity. Two validated composite indices were employed:
  ▪ DAS28 [6] integrates tender and swollen joint counts (out of 28 joints), the patient’s global assessment (visual analogue scale), and the ESR. Scores < 2.6 denote remission; 2.6–3.2, low activity; >3.2–5.1, moderate activity; and >5.1, high activity.
  ▪ RAPID3 [7] comprises patient-reported measures of pain, physical function, and global disease assessment, each on a 0–10 scale. Total scores ≤ 3 indicate remission; 3.01–6, low activity; 6.01–12, moderate activity; and >12, high activity.

### 2.5. Assessment of Health-Related Quality of Life

Health-related quality of life was assessed in both RA patients and controls using the SF-12 questionnaire [8]. This 12-item tool captures patient-reported perceptions of functional health and overall well-being, generating two composite indices: a Physical Component Summary (PCS) and a Mental Component Summary (MCS). As a streamlined adaptation of the SF-36, the SF-12 minimizes respondent burden while retaining robust psychometric properties. Each participant’s responses yielded a PCS and an MCS score, both scaled from 0 to 100, with higher values denoting better perceived health status.

### 2.6. SARC-F Screening and Complementary Measures

SARC-F was administered to both RA patients and controls. This questionnaire evaluates functional limitations related to muscle weakness through five items covering strength, walking ability, rising from a chair, climbing stairs, and history of falls. Each item is rated on a three-point scale, producing a total score ranging from 0 to 10, with values of 4 or higher indicating an increased likelihood of sarcopenia [4].

In addition, RA patients underwent an objective assessment of muscle strength and physical performance. Handgrip strength was assessed with a calibrated Jamar-type dynamometer (Kern 80K1). Each participant completed two attempts with both hands, and the highest measurement obtained from the dominant side was used for analysis. Low muscle strength was defined by the established cutoffs of <27 kg for men and <16 kg for women.

Gait speed was evaluated using a 6 m walk test. Participants were instructed to walk at their usual pace along a 6 m course, and the time required to complete the distance was recorded with a stopwatch to calculate speed (m/s). A value below 0.8 m/s was taken to indicate reduced physical performance.

### 2.7. Statistical Analysis

Given the exploratory design, no formal a priori sample size calculation was performed. We aimed to include a substantial proportion of eligible RA patients attending our clinic during the study period, together with age- and sex-matched controls, to ensure robust comparisons and preserve real-world applicability.

Continuous variables are described as mean with standard deviation or as median with interquartile range, whereas categorical data are summarized as counts and percentages. The distribution of continuous variables was examined using the Kolmogorov–Smirnov test. For comparisons between groups, we applied Student’s *t*-test or ANOVA when data followed a normal distribution, and Mann–Whitney U or Kruskal–Wallis tests otherwise. Categorical variables were analyzed using the χ^2^ test.

The primary outcome was the SARC-F score, analyzed both as a continuous variable and as a dichotomous variable (SARC-F ≥ 4). Comparisons of RA patients with controls were performed separately in men and women.

Bivariate correlations were used to assess the relationship between SARC-F scores and RA activity parameters (DAS28, RAPID3, ESR, and CRP). To identify independent correlates of SARC-F within the RA cohort, multivariable regression models were constructed. For SARC-F as a continuous variable, linear regression was applied; for SARC-F ≥ 4, logistic regression was used. Covariates included age, BMI, hemoglobin level, physical activity, RA disease duration, composite disease activity indices (DAS28, RAPID3), and current pharmacotherapy (glucocorticoids, conventional synthetic DMARDs, biologic DMARDs, and Janus kinase inhibitors).

All statistical tests were performed using a two-tailed approach, and significance was set at *p* < 0.05.

## 3. Results

We analyzed 575 individuals in total, comprising 275 patients with RA and 300 controls, all older than 50 years. Among the RA group, 191 were women (69.5%) and 84 were men (30.5%). An overview of their baseline demographic and clinical features is provided in Table 1.

The men were older than the women (71.9 ± 8.5 vs. 67.5 ± 8.8 years, *p* < 0.001) but had shorter disease duration (12.5 ± 9.6 vs. 16.8 ± 10.3 years, *p* < 0.01). No sex differences were observed for ESR, CRP, or autoantibody status. Women showed higher disease activity in both DAS28 (2.9 ± 1.1 vs. 2.5 ± 1.2, *p* < 0.01) and RAPID3 (9.7 ± 6.9 vs. 5.8 ± 5.5, *p* < 0.001), and poorer quality of life. In adjusted analyses, the SF-12 mental component remained significantly lower in women (−5.30, 95% CI −8.64 to −1.95, *p* < 0.01), while the physical component did not.

A SARC-F score ≥ 4 was observed in 26.9% of patients (74/275). Low grip strength was present in 52.4% of patients (144/275); of these, 61.1% (88/144) had a SARC-F score within the normal range. Overall, 20.4% of patients simultaneously presented a SARC-F score ≥ 4 and low grip strength, thereby fulfilling EWGSOP2 criteria for probable sarcopenia.

Low gait speed was observed in 22.5% of patients (62/275). Among them, 58.1% (36/62) also had a SARC-F score ≥ 4, and 48.4% (30/62) had concomitant low grip strength. The prevalence of abnormal SARC-F was higher in women (65/191, 34.0%) than in men (9/84, 10.7%). Sex-related differences were also observed in the proportion of patients with low grip strength (56.8% vs. 42.9%) and low gait speed (26.5% vs. 14.3%). After full adjustment, female sex remained an independent predictor of abnormal SARC-F (OR 3.14, 95% CI 1.24–7.95) along with RAPID3 (OR 1.25, 95% CI 1.18–1.33). In contrast, the associations between sex and low grip strength or low gait speed lost statistical significance once disease activity indices were included in the models. Sequential analyses suggested that RAPID3 and, to a lesser extent, DAS28 acted as major determinants of functional outcomes. RAPID3 explained much of the sex effect on SARC-F. For grip strength, DAS28 emerged as the key determinant, while age also contributed. For gait speed, the most consistent predictors were SARC-F itself and age, with DAS28 playing a smaller role.

In correlation analyses, SARC-F showed a moderate association with disease activity, more pronounced for RAPID3 than for DAS28. In women, SARC-F correlated with DAS28 (r = 0.33, *p* < 0.001) and RAPID3 (r = 0.59, *p* < 0.001), with only a weak correlation with ESR (r = 0.20, *p* < 0.05). In men, SARC-F also correlated with DAS28 (r = 0.35, *p* < 0.001) and RAPID3 (r = 0.72, *p* < 0.001), while no significant correlations were found with ESR or CRP in either sex.

Table 2 shows that men with RA did not differ from their age-matched controls in either median SARC-F values or in the proportion of individuals with scores ≥ 4. Among women with RA, however, both the median SARC-F score and the prevalence of abnormal scores (34.0% vs. 24.7%, *p* < 0.05) were significantly higher than in female controls.

## 4. Discussion

The aim of this study was to assess the performance of the SARC-F questionnaire in patients with RA, using age- and sex-matched controls, and to explore its clinical burden and correlates in routine practice. Our objective was to determine its utility for case-finding of sarcopenia in RA and to identify which patient profiles might benefit most from its use. To our knowledge, this is the first study in RA to address these questions within a control group design.

Mechanistically, chronic inflammation in RA promotes muscle wasting through several pathways. Pro-inflammatory cytokines such as TNF-α, IL-1β, and IL-6 stimulate muscle protein breakdown and inhibit muscle protein synthesis. In parallel, pain, functional limitation, reduced mobility, and cumulative glucocorticoid exposure may accelerate muscle loss by increasing catabolic signalling and limiting physical activity. Together, these mechanisms provide a biological basis for the increased burden of sarcopenia observed in RA [2,5].

Overall, nearly one-third of patients with RA had a SARC-F score ≥ 4. When compared with matched controls, this excess burden was evident only among women. In men, the frequency of abnormal SARC-F values did not differ from that of their matched controls, suggesting no substantial increase in sarcopenia risk beyond the background population. In contrast, women with RA had significantly higher scores and a markedly greater prevalence of abnormal results, identifying them as a target group in whom SARC-F case finding is especially warranted.

In our cohort, men were on average older but had a shorter disease duration, whereas women were younger yet had accumulated longer exposure to RA. This pattern has been consistently described in other series [9,10], reflecting the earlier onset of RA in women. Importantly, our analyses were adjusted for age and the excess burden of abnormal SARC-F in women persisted. Thus, the observed sex difference in sarcopenia risk cannot be attributed to demographic imbalance alone but represents an independent association.

Our findings are consistent with previous reports showing that women with RA experience greater disease burden and worse patient-reported outcomes than men, despite comparable levels of objective inflammation. Large cohorts such as QUEST-RA [11] and registry-based studies such as BIOBADASER III [12] have described higher disability, poorer quality of life, and greater pain and fatigue among women, in line with our observation that sex disparities were most pronounced in patient-reported and functional measures, including SARC-F. The convergence of our results with these studies suggests that the excess sarcopenia risk identified by SARC-F is part of a broader and well-documented pattern of sex-related vulnerability in RA.

The independent association between female sex and abnormal SARC-F scores is particularly noteworthy. In our cohort, women were more than three times as likely as men to reach the frailty threshold, a difference that persisted after adjustment for demographic and disease-related variables. This excess risk may partly reflect factors not fully captured in our study, such as lower baseline muscle mass and strength in women, longer cumulative disease exposure, or a greater burden of pain and fatigue that may limit physical activity. The persistence of poorer SF-12 mental health scores in women further suggests that psychological and social dimensions could also contribute to frailty risk as identified by SARC-F.

When associations with clinical parameters were examined, marked sex-specific patterns emerged. Female sex and RAPID3 were independent determinants of abnormal SARC-F. This suggests that in women, SARC-F reflects a broader vulnerability that overlaps with—but is not fully explained by—disease activity or health-related quality of life.

Consistent with this interpretation, SARC-F showed only weak or absent associations with inflammatory markers, while correlating more strongly with disease activity—particularly RAPID3—supporting that it primarily reflects the functional and patient-perceived burden of RA rather than isolated inflammation.

Beyond prevalence, our study also contributes to clarifying the relationship between SARC-F and objective measures of sarcopenia. More than half of our patients presented low grip strength, and within this group a relevant proportion had SARC-F values within the normal range. This highlights that although SARC-F is valuable as a first-line screening tool, it does not capture all cases of functional impairment. Handgrip strength therefore remains essential, not only as the cornerstone of the EWGSOP2 framework but also as an independent predictor of sarcopenia, disability, and mortality across populations [13,14].

The integration of SARC-F with objective strength measures provides additional insight. Patients with both SARC-F ≥ 4 and low grip strength fulfilled the EWGSOP2 definition of probable sarcopenia and represented a sizeable proportion of our cohort, highlighting the potential clinical relevance of combining these assessments. Published RA cohorts [15,16,17,18] that applied the full EWGSOP2 algorithm have reported prevalences of confirmed sarcopenia ranging from 4.5% to 19%, depending on age distribution and sex composition, while figures for probable sarcopenia are typically higher. Considering both our findings and published data, a pragmatic approach based on SARC-F complemented with grip strength might be sufficient to identify most RA women at risk.

In our cohort, one in five patients also showed reduced gait speed. Although gait speed represents the final step toward severe sarcopenia in EWGSOP2, its interpretation in RA is less straightforward, as joint pain, stiffness, and disability may contribute independently to muscle function. Nonetheless, the overlap we observed between slow gait, abnormal SARC-F, and low grip strength supports the internal consistency of our findings and suggests that performance-based measures may add complementary value in selected patients.

Handgrip dynamometry is the reference measure of muscle strength in EWGSOP2; in RA, however, results may be confounded in patients with severe hand involvement (marked activity or structural damage). In such cases, complementary lower-limb tests such as the five-times sit-to-stand can provide a more reliable estimate of overall strength and help avoid misclassification. Confirmation of muscle mass by DXA or BIA may be reserved for situations in which a definitive diagnostic label would alter management.

From a clinical perspective, rheumatologists should incorporate SARC-F as an initial case-finding tool and systematically assess handgrip strength in patients with suspected sarcopenia. Gait speed can provide complementary information in patients with marked functional impairment. Monitoring these parameters, alongside disease activity and physical performance, may facilitate earlier identification and targeted intervention in routine practice.

This study has limitations. First, muscle mass was not assessed with DXA or BIA, preventing definitive EWGSOP2 classification; instead, we focused on SARC-F and muscle strength, the two initial steps of the EWGSOP2 algorithm most relevant for pragmatic implementation. Second, controls were recruited from diverse hospital-based and community sources, which may have introduced heterogeneity despite careful matching and exclusion of conditions associated with secondary sarcopenia. Third, the cross-sectional design precludes causal inference, and the prognostic significance of abnormal SARC-F in RA requires confirmation in longitudinal studies. Finally, as an exploratory study, larger confirmatory cohorts will be needed to formally validate the screening performance of SARC-F in RA.

Nonetheless, our study also has important strengths. To the best of our knowledge, this is the first study in RA to analyze SARC-F within a control group framework, allowing the prevalence findings to be interpreted in relation to a matched reference population. We combined self-reported and objective measures, integrating PROM-based screening (SARC-F) with grip strength and gait speed, thereby ensuring internal consistency and clinical applicability. The use of multivariable models further reinforced the robustness of our findings, showing that the excess risk associated with female sex persisted after adjustment for demographic and disease-related variables. Finally, the focus on sex-stratified analyses allowed a more nuanced interpretation, revealing that the burden of abnormal SARC-F values lies primarily among women. Together, these strengths enhance the validity and clinical relevance of our results.

In conclusion, our study shows that the SARC-F questionnaire identifies a substantial burden of sarcopenia risk in RA, with the excess concentrated among women. When complemented by simple grip strength testing, it provides a pragmatic and feasible strategy for case finding in routine rheumatology practice, even in settings without access to advanced body composition techniques. This approach facilitates timely recognition of vulnerable patients and supports preventive strategies such as exercise and nutritional interventions. Incorporating SARC-F into RA care pathways may represent a low-cost, patient-centred step toward addressing an overlooked comorbidity. Future longitudinal studies should confirm its prognostic value and clarify the impact of targeted interventions in this high-risk population.

## Figures and Tables

**Table 1:** Characteristics of the 275 RA patients, stratified by sex into men (n = 84) and women (n = 191). Association with the study variables.

	Men	Women	Unadjusted Parameter Estimate (95% CI)	*p*-Value	Adjusted * Parameter Estimate (95% CI)	*p*-Value
Age (years)	71.9 ± 8.5	67.5 ± 8.8	−4.45 (−6.71, −2.19)	<0.001	-	-
BMI (kg/m^2^)	27.5 ± 3.5	27.9 ± 5.4	-	ns	-	-
Underweight (n, %)	1 (1.2%)	0	-	ns	-	-
Normal range (n, %)	18 (21.4%)	64 (33.5%)	R	-	-	-
Overweight (n, %)	49 (58.3%)	72 (37.5%)	2.42 (1.28, 4.57)	<0.01	2.44 (1.28, 4.69)	<0.01
Obese (n, %)	16 (19.1%)	55 (29%)	-	-	-	-
Smoking						
Never (n, %)	72 (85.7%)	164 (87.2%)	-	ns	-	-
Former (n, %)	1 (1.2%)	2 (1.1%)				
Current (n, %)	11 (13.1%)	22 (11.7%)				
Physical activity						
None (n, %)	31 (37%)	94 (50.3%)	-	ns	-	-
Sporadic (n, %)	16 (19%)	36 (19.3%)				
Regular with low intensity (n, %)	33 (39.2%)	55 (29.4%)				
Regular with high intensity (n, %)	4 (4.8%)	2 (1.1%)				
Hemoglobin (g/dL)	14.2 ± 1.5	13.3 ± 1.2	−0.84 (−1.16, −0.52)	<0.001	−1.01 (−1.33, −0.69)	<0.001
Disease duration (years)	12.5 ± 9.6	16.8 ± 10.3	3.86 (1.16, 6.57)	<0.01	4.61 (1.84, 7.37)	<0.01
RF + (n, %)	50/83 (60.2%)	123/168 (73%)	-	ns	-	-
RF titer (UI/L)	175 ± 224	208 ± 415	-	ns	-	-
ACPA + (n, %)	52/83 (62.6%)	115/167 (69%)	-	ns	-	-
ACPA titer (U/L)	571 ± 1040	369 ± 672	-	ns	-	-
Current medication						
Glucocorticoids (n, %)	46 (54.7%)	89 (46.5%)	-	ns		ns
cDMARDs (n, %)	73 (86.9%)	172 (90%)	-	ns	-	ns
bDMARDs (n, %)	20 (23.8%)	68 (36%)	0.75 (0, 2.11)	<0.001	-	ns
Jak inhibitors (n, %)	2 (2.4%)	10 (5%)	-	ns	-	ns
ESR (mm/h)	24.3 ± 26.2	24.8 ± 20.8	-	ns	-	-
CRP (mg/dL)	10.1 ± 18.3	5.2 ± 6.1	−3.67 (−7.16, −0.17)	<0.05	−6.30 (−10.64, −2.0)	<0.01
DAS28	2.5 ± 1.2	2.9 ± 1.1	0.46 (0.16, 0.75)	<0.01	0.49 (0.13, 0.84)	<0.01
Remission (n, %)	49 (58.3%)	77 (40.5%)	R	ns	R	-
LDA (n, %)	16 (19%)	47 (24.5%)	-	ns	-	ns
MDA (n, %)	15 (17.9%)	61 (32%)	0.39 (0.20, 0.75)	<0.01	0.37 (0.14, 0.90)	<0.05
HDA (n, %)	4 (4.8%)	6 (3%)	-	ns	-	ns
RAPID3	5.8 ± 5.5	9.7 ± 6.9	3.59 (1.78, 5.40)	<0.001	2.46 (0.63, 4.28)	<0.01
Remission (n, %)	37 (44%)	38 (24%)	R	-	R	-
LDA (n, %)	11 (13.1%)	13 (8%)	-	ns	-	ns
MDA (n, %)	27 (32.1%)	55 (34%)	-	ns	-	ns
HDA (n, %)	9 (10.8%)	55 (34%)	0.19 (0.08, 0.45)	<0.001	0.24 (0.07, 0.76)	<0.05
SF-12						
Mental health	51.5 ± 10.0	45.1 ± 11.4	−6.46 (−9.35, −3.56)	<0.001	−5.30 (−8.64, −1.95)	<0.01
Physical health	42.5 ± 9.6	36.8 ± 9.5	−5.71 (−8.22, −3.20)	<0.001	-	ns
SARC-F (median, interquartilic range)	1 [0–2]	2 [1–4]	1.10 (0.75, 1.45)	<0.001	0.87 (0.41, 1.34)	<0.001
SARC-F ≥ 4 (n, %)	9 (10.7%)	65 (34.0%)	2.76 (1.71, 4.47)	<0.001	3.14 (1.24, 7.95)	<0.05
Low grip strength (n, %)	36 (42.9%)	108 (56.8%)	1.76 (1.05, 2.95)	<0.05	-	ns
Low gait speed (n, %)	12 (14.3%)	50 (26.5%)	2.16 (1.08, 4.31)	<0.05	-	ns

* Each analysis was adjusted for all variables that were found to be significant in the previous rows of the table. CI, confidence interval; BMI, body mass index; R, reference category; RF, rheumatoid factor; ACPAs, anti-citrullinated peptide antibodies; ESR, erythrocyte sedimentation rate; CRP, C-reactive protein; DAS28, Disease Activity Score 28; LDA, low disease activity; MDA, moderate disease activity; HDA: high disease activity; RAPID3, Routine Assessment of Patient Index Data 3; HAQ, Health Assessment Questionnaire; cDMARDs, conventional disease-modifying antirheumatic drugs; bDMARDs; biological disease-modifying antirheumatic drugs; IQR, interquartile range; SF-12: Short Form Health Survey-12.

**Table 2:** Comparison of demographic and clinical characteristics between male and female patients with rheumatoid arthritis RA, and between each sex-specific RA group and their respective age-matched controls.

	Male			Female		
	Patients (n: 84)	Controls (n: 102)	*p*	Patients (n: 191)	Controls (n: 198)	*p*
Age (years)	71.9 ± 8.6	71.1 ± 9.2	ns	67.5 ± 8.8	67.3 ± 9.2	ns
BMI (kg/m^2^)	27.5 ± 3.5	27.4 ± 4.4	ns	27.9 ± 5.4	27.8 ± 5.3	ns
Underweight (n, %)	1 (1.2%)	0		0	4 (2%)	
Normal range (n, %)	18 (21.4%)	33 (32.3%)		64 (34%)	57 (30%)	
Overweight (n, %)	49 (58.3%)	46 (45.1%)		72 (37%)	73 (38%)	
Obese (n, %)	16 (19.1%)	23 (22.6%)	ns	55 (29%)	60 (30%)	ns
Hemoglobin (g/dL)	14.2 ± 1.4	14.5 ± 1.6	ns	13.3 ± 1.2	13.7 ± 1.1	<0.01
SF-12						
Mental health	51.5 ± 10.0	50.8 ± 9.9	ns	45.1 ± 11.4	50.2 ± 10.1	<0.001
Physical health	42.5 ± 9.6	46.7 ± 10.6	<0.01	36.8 ± 9.5	44.0 ± 11.5	<0.001
SARC-F (median, interquartilic range)	1 [0–2]	0 [0–2]	ns	2 [1–4]	1 [0–3.25]	<0.001
SARC-F ≥ 4 (n, %)	9 (10.7%)	15 (15.0%)	ns	65 (34.0%)	49 (24.7%)	<0.05

BMI, body mass index; SF-12, Short Form Health Survey-12.

## Data Availability

The datasets used and analyzed during the current study are available from the corresponding author upon reasonable request.
